# Response of bacterial community structure to different phosphorus additions in a tobacco-growing soil

**DOI:** 10.3389/fpls.2024.1344733

**Published:** 2024-03-07

**Authors:** Beibei Zhou, Shiqian Cheng, Shuang Peng, Wenqing Li, Chunying Li, Qianqian Wang, Yiming Wang, Jinping Guo

**Affiliations:** ^1^ School of Environment and Ecology, Jiangsu Open University, Nanjing, China; ^2^ State Key Laboratory of Soil and Sustainable Agriculture, Institute of Soil Science, Chinese Academy of Sciences, Nanjing, China; ^3^ School of Agriculture, Ningxia University, Yinchuan, China; ^4^ Fujian Tobacco Research Institute, Fuzhou, China

**Keywords:** tobacco growth, phosphorus addition, high-throughput sequencing, bacterial community, soil P transformation

## Abstract

**Introduction:**

Phosphorus (P), which plays a vital role in plant growth, is continually added to soil to maximize biomass production, leading to excessive P accumulation and water eutrophication.

**Results:**

In this study, a pot experiment using a subtropical tobacco-growing soil fertilized with four P levels—no P, low P, medium P, and high P—was conducted and rhizosphere and bulk soils were analyzed.

**Results:**

P addition significantly increased tobacco biomass production (except under low P input) and total soil P and available P content (*P*<0.05), whereas total nitrogen content decreased in the rhizosphere soils, although this was only significant with medium P application. P fertilization also significantly altered the bacterial communities of rhizosphere soils (*P*<0.05), but those of bulk soils were unchanged (*P*>0.05). Moreover, a significant difference was found between rhizosphere soils with low (LR) and high (HR) P inputs (*P*<0.05). Additionally, compared with rhizosphere soils with no P (CKR), Shannon diversity showed a declining trend, which was significant with LR and HR (*P*<0.05), whereas an increasing tendency was observed for Chao1 diversity except in LR (*P*>0.05). Functional prediction revealed that P application significantly decreased the total P and N metabolism of microorganisms in rhizosphere soils (*P*<0.05).

**Discussion:**

Collectively, our results indicate that maintaining sustainable agricultural ecosystems under surplus P conditions requires more attention to be directed toward motivating the potential of soil functional microbes in P cycling, rather than just through continual P input.

## 1 Introduction

Phosphorus (P) is an essential macronutrient for plants, playing a crucial role in the synthesis of nucleic acids, enzymes, and coenzymes (Mehnaz et al., 2019). However, P utilization in agroecosystems is always inefficient (10%–20%), making P a major limiting nutrient in agriculture (Pantigoso et al., 2020; Liu et al., 2022). After being applied to soils, P fertilizers are easily absorbed and fixed, thus the P is inaccessible to plants. Consequently, to guarantee crop yields, the continual application of P fertilizer in large amounts is a common agronomic measure; however, this measure accelerates the depletion of non-renewable P resources and leads to excessively high soil P levels, threatening water quality (Cahoon and Ensign, 2004; Liu et al., 2018). Thus, facilitating soil P cycling and improving P availability remain high priorities in sustainable agricultural management (Huang et al., 2021).

Microorganisms are actively involved in regulating soil P cycling and increasing soil P availability (Richardson and Simpson, 2011; Zheng et al., 2018). In soils, P exists in organic (Po) and inorganic (Pi) forms, but only the inorganic orthophosphate anions (PO_4_^3−^) are available to plants (Lagos et al., 2016). Microbes can access and recycle P by secreting organic acids to solubilize Pi and releasing phosphatases, such as acid phosphatase (ACP), alkaline phosphatase (ALP), and phytase, to mineralize Po (Azene et al., 2023). *Pseudomonas*, *Burkholderia*, *Bacillus*, and *Rhizobium* are bacterial genera that are recognized for their capacity to mobilize recalcitrant P (Browne et al., 2009; Liu et al., 2022). Studies have revealed that differential P fertilization regimes affect soil bacterial community composition, diversity, and the corresponding functional profiles, and subsequently impact P transformation behaviors (Lagos et al., 2016; Liu et al., 2018; Pantigoso et al., 2020). However, contradictory effects of P addition on soil bacterial diversity and community structures are often reported, which may be ascribed to different P fertilization rates (Sun et al., 2022), crop rotation (Pantigoso et al., 2020), soil fertility (Heuck et al., 2015; Spohn et al., 2018), and land use (Wu et al., 2022). Lagos et al. (2016) found that the addition of P significantly altered the rhizobacterial community composition of ryegrass, whereas a study by Liu et al. (2018) reported no significant effects on the community and diversity of soil bacteria under P surplus conditions.

Tobacco (*Nicotiana tabacum*) is an important economic crop in China, with an annual planting area of approximately 1.41 million hectares (Zheng et al., 2020). As one of the major tobacco producing areas, Fujian Province has experienced long-term tobacco cultivation that has resulted in excessively high soil P levels, with an average concentration of 50.46 mg/kg (Tang et al., 2022). However, although P is often highly concentrated in the tobacco-growing soil, only a small component is directly available to plants, as the tobacco plants are usually short after transplanting with no extra P fertilizer, indicating that the early tobacco growth is limited by P (Xie et al., 2017). Similarly, to maximize tobacco biomass production, continual anthropogenic P inputs are inevitable (Tang et al., 2022), which is unfriendly to soil fertility and surface waters. Therefore, a comprehensive understanding of the responses of tobacco plants and the physicochemical properties of soil to P addition, as well as the relevant key bacteria, is vital for tobacco production, soil sustainability, and water protection. Although related studies have been conducted in other plants (Ling et al., 2017; Pantigoso et al., 2020), to our knowledge, there is limited research in tobacco, and the relevant conclusions may vary depending on the plant species. Hence, in this study, a pot experiment was conducted with tobacco plants under four simulated P input rates. High-throughput sequencing of bacterial 16S rRNA genes was performed to obtain information on the bacterial composition and diversity under P addition. The main objectives of the study were to elucidate: (1) the effects of different P input rates on tobacco growth and biomass accumulation; (2) the influence of P addition on soil properties; and (3) the responses of soil bacterial communities and diversity, and the functional traits regarding P and N cycles, to different P inputs. The results of this study contribute to improving our capacity to predict how a soil bacterial community will respond to continual P input in tobacco-growing regions and developing effective strategies for maintaining the sustainability of agroecosystems under nutrient additions.

## 2 Materials and methods

### 2.1 Greenhouse experiment design

The P input pot experiment was conducted in a greenhouse at the Institute of Soil Science, Chinese Academy of Sciences. Four P input rates—0 g P_2_O_5_ pot^−1^ (CK), 1.35 g P_2_O_5_ pot^−1^ (L), 2.7 g P_2_O_5_ pot^−1^ (M) and 5.4 g P_2_O_5_ pot^−1^ (H) —were applied to the soil in the form of KH_2_PO_4_. In addition, all four treatments were applied with the same amount of nitrogen (KNO_3_ and urea) and potassium (KNO_3_). Three replicates were set up for each treatment and 5 kg of air-dried soil was transferred to each plastic pot. The soil was subtropical paddy soil collected from Longyan in Fujian Province, China (116°30′ E, 24°56′ N) and contained 1.43 g/kg total N (TN), 0.59 g/kg total P (TP), 8.92 g/kg total K, and 41.72 mg/kg available P (AP), with a pH of 6.28. After mixing the soil and fertilizer, a 55-day-old tobacco seedling was transplanted to each pot on December 15, 2022. Soil and tobacco plant samples were collected on January 12, 2023. For soil sampling, after the tobacco was harvested, the roots with attached soil were removed from the pots. Soil loosely adhering to the roots was removed and the remaining attached soil was defined as rhizosphere soil (RS). The remaining soil in each pot was sampled as bulk soil (BS).

### 2.2 Analyses of soil and plants

Soil pH was measured in a 1:2.5 (soil:water) mixture using a pH meter. Measurement of the following soil properties was conducted according to Lu (1982). Soil TP was determined using the molybdenum blue colorimetric method after digestion with H_2_SO_4_-HClO_4_. Soil TN and available N (AN) were measured using semi-micro Kjeldahl and the alkaline hydrolysis diffusion method, respectively. Soil AP was extracted with 0.5 mol L^−1^ NaHCO_3_. Soil N:P and phosphorus activation coefficient (PAC) were calculated by dividing the concentrations of TN and AP by TP, respectively. Soil P fractionation, including Al-P, Fe-P, Ca-P, and O-P, was measured using the method described by (Hedley et al. (1982). Phosphatase enzymes, including ACP, ALP, and phytase, were determined using commercially available kits (Beijing, Solarbio Company).

The leaves of each harvested tobacco plant were weighed after drying to a constant weight. Plant TN and TP contents were determined using semi-micro Kjeldahl and the vanadium molybdate yellow colorimetric method, respectively, after digestion with H_2_SO_4_-H_2_O_2_ (Lu, 1982).

### 2.3 DNA extraction and sequencing of 16S rRNA gene amplicons

Total genomic DNA was extracted from 0.5 g of moist soil using the FastDNA^®^ SPIN Kit for soil (MP Biomedicals, Santa Ana, CA, USA) according to the manufacturer’s instructions. The quality and quantity of the extracted DNA were checked using a NanoDrop ND-1000 spectrophotometer (Thermo Scientific, Waltham, MA, USA), and the DNA was stored at −20°C until use. The bacterial 16S rRNA gene was amplified using the primer set 515F/806R (Wang et al., 2021) and sequenced on an Illumina NovaSeq platform (Novogene, Beijing, China). Raw bacterial 16S rRNA gene data were processed using the Quantitative Insights Into Microbial Ecology (QIIME) bioinformatics platform according to Caporaso et al. (2010). After quality filtering and denoising, the sequences were binned into operational taxonomic units (OTUs) using a 97% identity threshold. The Silva database was then used to annotate taxonomic information for the representative sequence of each OTU (Quast et al., 2012). Subsequently, sequences were submitted to the National Center for Biotechnology Information (NCBI) database under accession number SRP468041.

### 2.4 Statistical analyses

Statistical analyses were performed using SPSS v 13.0 (SPSS Inc., Chicago, IL, USA). Data were expressed as the mean ± standard deviation (SD). Letters above the error bars in the figures and following the mean ± SD values in the tables indicate significant differences between treatments. Mean separation was assessed by Tukey’s test. Differences at *P*<0.05 were considered statistically significant.

PICRUSt2 was used to predict functional enzymes related to N and P metabolism based on 16S rRNA gene data with the Kyoto Encyclopedia of Genes and Genomes (KEGG) database, as described by Wang et al. (2021). A co-occurrence network between P input rates and bacterial genera was constructed according to the Pearson’s correlation coefficients (r > 0.6 or r < −0.6) and *P* value (*P* < 0.05) and visualized using Gephi software (Ling et al., 2017). Partial least squares path modeling (PLS-PM) was performed to infer the potential direct and indirect effects of P input rate, soil properties, bacterial composition, bacterial function, and bacterial diversity on tobacco growth. The quality of the PLS-PM is evaluated by the goodness of fit (GOF) index, with a GOF index >0.7 indicating a good overall prediction performance of the model, and R^2^ values represent the variance of dependent variables explained by the inner model (Tian et al., 2022).

## 3 Results

### 3.1 Analysis of plant and soil properties

Compared with CK, P application increased tobacco growth (**Table 1**), and this effect was significant at medium and high P input levels (*P*<0.05). P application also promoted N uptake by tobacco, especially at the medium application rate. With the increase in the P application rate, the P concentration of tobacco also increased.

**Table 1:** Biomass and nutrient contents of tobacco as well as the soil chemical properties and enzyme activities.

		CK		L		M		H	
Plant properties	Biomass (g)	1.63 ± 0.07c		2.14 ± 0.18bc		2.25 ± 0.14b		2.88 ± 0.31a	
	N content (g/kg)	43.40 ± 2.60b		47.75 ± 1.94ab		48.82 ± 2.01a		48.38 ± 3.22ab	
	P content (g/kg)	1.18 ± 0.07d		2.33 ± 0.20c		3.05 ± 0.11b		3.77 ± 0.15a	
		RS	BS	RS	BS	RS	BS	RS	BS
Soil chemical properties	pH	5.72 ± 0.09a	5.67 ± 0.03a	5.74 ± 0.03a	5.65 ± 0.05a	5.73 ± 0.05a	5.63 ± 0.04a	5.70 ± 0.11a	5.59 ± 0.02a
	TN (g/kg)	1.81 ± 0.01a	1.72 ± 0.09ab	1.68 ± 0.06ab	1.79 ± 0.05ab	1.63 ± 0.04b	1.82 ± 0.06a	1.78 ± 0.07ab	1.79 ± 0.06ab
	TP (g/kg)	0.58 ± 0.02e	0.59 ± 0.01e	0.76 ± 0.03cd	0.73 ± 0.06d	0.89 ± 0.02b	0.86 ± 0.02bc	1.15 ± 0.05a	1.17 ± 0.10a
	AN (g/kg)	0.18 ± 0.02c	0.22 ± 0.01abc	0.23 ± 0.01ab	0.21 ± 0.02abc	0.22 ± 0.01abc	0.22 ± 0.02abc	0.19 ± 0.03bc	0.24 ± 0.01a
	AP (mg/kg)	39.33 ± 1.90d	43.43 ± 3.40d	69.76 ± 0.59bc	70.54 ± 3.92c	88.23 ± 3.34b	86.27 ± 0.936b	115.23 ± 1.68a	116.08 ± 3.11a
	N:P	3.11 ± 0.24a	2.91 ± 0.18a	2.16 ± 0.04bc	2.46 ± 0.23b	1.83 ± 0.07cd	2.13 ± 0.06bc	1.55 ± 0.03d	1.49 ± 0.11d
	PAC (%)	6.81 ± 0.15a	7.36 ± 0.59ab	9.17 ± 0.43ab	9.75 ± 1.20ab	9.88 ± 0.14b	10.08 ± 0.28a	10.07 ± 0.38ab	9.98 ± 0.97ab
Soil enzymes	ALP (U/g)	5375 ± 258bc	6057 ± 258b	4634 ± 278c	7333 ± 655a	5900 ± 239b	5392 ± 257bc	5912 ± 386b	5909 ± 195b
	ACP (U/g)	47827 ± 4061a	53945 ± 3670a	51378 ± 4187a	49788 ± 2076a	49777 ± 4179a	53179 ± 3867a	47399 ± 1856a	53358 ± 5173a
	Phytase (U/g)	7.39 ± 0.69d	7.23 ± 0.41d	11.88 ± 1.06c	12.40 ± 0.46c	14.68 ± 0.96bc	15.78 ± 1.23b	24.68 ± 1.83a	22.49 ± 1.09a

Data are means ± standard deviation. Different letters followed the values indicate significant differences (P<0.05). CK, no P fertilization; L, low P input rate; M, medium P input rate; H, high P input rate. RS and BS represent rhizosphere and bulk soils, respectively.

Regarding changes in soil physicochemical parameters, P application decreased rhizosphere soil TN content, and this was significant at the medium application rate (*P*<0.05). For soil TP and AP, P application significantly increased their contents (*P*<0.05), but no significant difference was found between rhizosphere and bulk soils (*P*>0.05). The same trends were observed for N:P and PAC, but the degree of deviation varied among treatments.

With respect to soil phosphatase activity, no significant difference was observed between rhizosphere and bulk soils (*P*>0.05), except for ALP under low P input, with the minimum and maximum activities occurring in the rhizosphere soil and bulk soil, respectively. There was also no significant difference in ACP among the four treatments (*P*>0.05), but at the low P application rate, ACP activity in the rhizosphere soil was greater than that in the bulk soil, contrary to the performance of ALP. With the increase of P application, soil phytase activity increased.

P fractionation of rhizosphere and bulk soils was performed, and the results are presented in **Table 2**. Fe-P was the dominant P form, occupying more than 50%, followed by Al-P. There was no significant difference between rhizosphere and bulk soils in the relative abundance of Fe-P (*P*>0.05), which significantly increased with the ascending P application. The same trend of increasing content with increasing P application rate was found with Al-P, but at the high P application, the rhizosphere soil contained significantly more Al-P (*P*<0.05). The contents of Ca-P and O-P were very low, and no significant differences were detected among all soils (*P*>0.05).

**Table 2:** Sequentially-extracted P fractions in the rhizosphere and bulk soils of different treatment.

		CK		L		M		H	
		RS	BS	RS	BS	RS	BS	RS	BS
Content (mg/kg)	Al-P	53.20 ± 3.82e	60.33 ± 3.27e	112.09 ± 2.80d	127.73 ± 18.65cd	139.00 ± 12.11cd	159.70 ± 12.25c	331.15 ± 26.57a	236.62 ± 38.38b
	Fe-P	217.22 ± 13.82d	226.87 ± 2.58d	307.96 ± 13.66c	303.28 ± 5.16c	353.54 ± 33.88b	357.76 ± 9.51b	435.71 ± 21.04a	474.09 ± 29.79a
	Ca-P	25.03 ± 0.83a	25.74 ± 3.00a	26.24 ± 3.72a	30.83 ± 3.44a	26.68 ± 2.67a	25.95 ± 1.59a	27.22 ± 3.24a	24.48 ± 3.45a
	O-P	23.01 ± 5.51a	11.84 ± 3.42a	17.88 ± 5.030a	11.84 ± 2.41a	27.13 ± 5.64a	17.04 ± 4.88a	17.28 ± 8.00a	23.04 ± 13.68a
Relative Content (%)	Al-P_r_	16.71 ± 0.63d	17.06 ± 2.36d	24.17 ± 0.74c	26.31 ± 2.06bc	25.51 ± 2.22cd	28.03 ± 1.66bc	39.47 ± 3.78a	31.36 ± 4.12b
	Fe-P_r_	68.21 ± 1.43a	63.98 ± 6.00ab	66.34 ± 1.08ab	62.97 ± 5.19abc	64.67 ± 2.47ab	62.88 ± 3.01abc	54.96 ± 3.62c	58.72 ± 3.25bc
	Ca-P_r_	7.89 ± 0.62a	6.70 ± 0.64ab	5.66 ± 0.87bc	6.36 ± 0.29c	4.90 ± 0.50c	4.56 ± 0.30cd	3.43 ± 0.40de	2.84 ± 0.50e
	O-P_r_	7.20 ± 1.49a	3.58 ± 1.04bc	3.83 ± 0.97bc	2.55 ± 0.52bc	4.93 ± 0.70ab	3.00 ± 0.80bc	2.14 ± 0.87c	2.78 ± 1.37bc

Data are means ± standard deviation. Different letters followed the values indicate significant differences (P<0.05). CK, no P fertilization; L, low P input rate; M, medium P input rate; H, high P input rate. RS and BS represent rhizosphere and bulk soils, respectively.

### 3.2 Effects of different P inputs on soil bacterial community composition

**Figure 1:**
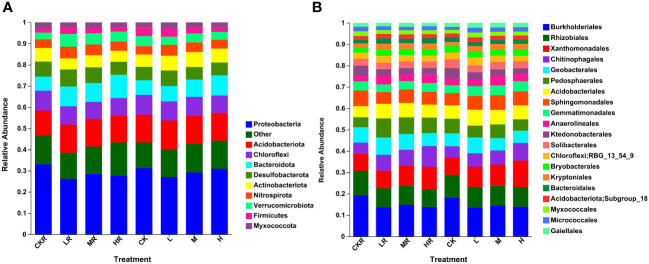
Relative abundance of soil bacteria at phylum **(A)** and order **(B)** levels with different phosphorous (P) treatments. CK, no P fertilization; L, low P input rate; M, medium P input rate; H, high P input rate. R represents the rhizosphere soil.

As shown in **Figure 1A**, the top ten abundant bacterial phyla were Proteobacteria (26.16–33.06%), Acidobacteriota (11.85–13.63%), Chloroflexi (7.97–9.39%), Bacteroidota (6.63–11.02%), Desulfobacterota (5.92–8.06%), Actinobacteriota (5.18–7.06%), Nitrospirota (3.99–5.53%), Verrucomicrobiota (3.25–5.96%), Firmicutes (1.96–4.18%), and Myxococcota (2.25–2.91%). Significant differences in the relative abundance of Proteobacteria were found among rhizosphere soils, with the proportion decreasing significantly in the soils with the low (LR) and high (HR) P application rates relative to no P treatment (CKR) (*P*<0.05). For bulk soils, there was no significant difference in the relative abundance of Proteobacteria among the different treatments. The abundance of Bacteroidota in LR (*P*<0.05) and HR (*P*<0.001) samples was significantly higher than that of CKR, which was also true for the bulk soil at a high P application rate (H) (*P*<0.05). The rhizosphere soils contained more Desulfobacterota than the bulk soils, with a significant difference between bulk soil with medium P input (M) and rhizosphere soil with medium P input (MR) (*P*<0.05). However, under high P application rates, the relative abundance of Desulfobacterota was almost the same in rhizosphere and bulk soils. Moreover, the relative abundance of Desulfobacterota in HR was significantly lower than that in the other three rhizosphere soils (*P*<0.05). For all treatments, the abundance of Nitrospirota was higher in the rhizosphere soil than in the bulk soil, although the difference was not significant, and the abundance was significantly increased in LR and MR relative to HR (*P*<0.05). In contrast to Desulfobacterota and Nitrospirota, the abundance of Firmicutes was higher in the bulk soils than in the rhizosphere soils except under high P application.

**Figure 1B** illustrates the relative abundance of the top 20 bacterial orders of different treatments, with the predominant orders being Burkholderiales (7.10–10.71%), Rhizobiales (4.60–6.39%), Xanthomonadales (4.39–7.02%), Chitinophagales (2.93–5.34%), Geobacterales (3.15–4.38%), Pedosphaerales (2.61–4.87%), Acidobacteriales (2.89–3.91%), and Sphingomonadales (2.98–4.07%). P application significantly decreased the abundance of Burkholderiales in rhizosphere and bulk soils (*P*<0.05). A decrease was also observed for Rhizobiales, but the difference was not significant in the bulk soils (*P*>0.05). Under the condition of high P input, the abundance of Chitinophagales was increased and was significant relative to CK for rhizosphere and bulk soils (*P*<0.05). In addition, the relative abundance of Geobacterales was increased at a low P application rate and a significant difference was detected between LR and HR (*P*<0.05).

### 3.3 Effects of different P inputs on soil bacterial diversity

**Figure 2:**
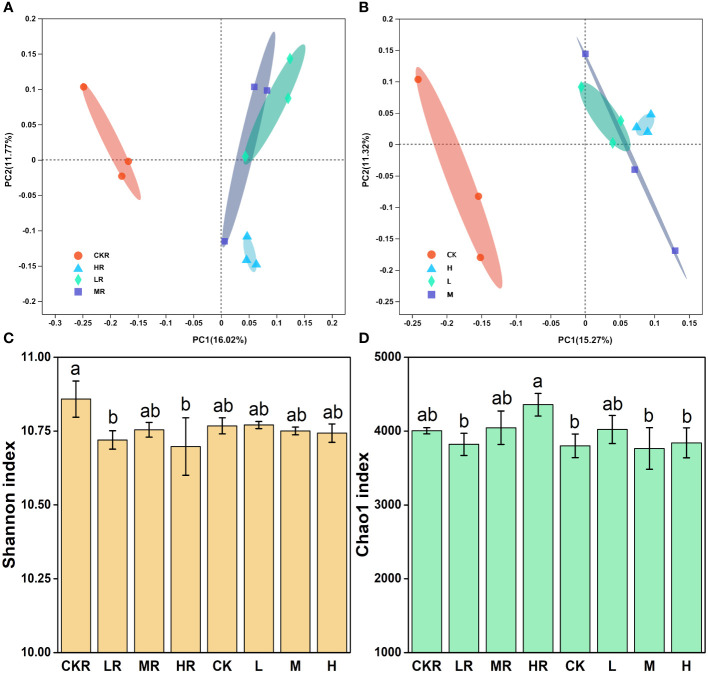
Effects of different P addition rates on bacterial community composition in the rhizosphere **(A)** and bulk **(B)** soils, as well as on bacterial diversity **(C)** and richness **(D)**. CK, no P fertilization; L, low P input rate; M, medium P input rate; H, high P input rate. R represents rhizosphere soil. Different letters above the error bars indicate significant differences (*P<*0.05).

Principal coordinate analysis (PCoA) revealed that P application altered the rhizosphere (**Figure 2A**) and bulk (**Figure 2B**) soil bacterial communities of tobacco relative to no P treatment (CK), as samples of the P-amended soils clustered together, dissimilating from those of the soils with no P. The difference in soil bacterial community was significant between CKR and the three P added treatments in rhizosphere soil (LR: *P*=0.039, MR: *P*=0.017, HR: *P*=0.022), but was not significant among bulk soils according to the results of adonis analysis. Furthermore, the addition of P at three loading rates did not significantly alter bacterial communities for the rhizosphere and bulk soils, excluding HR and LR (*P*<0.05).

There was no significant difference in the Shannon (**Figure 2C**) and Chao1 (**Figure 2D**) indices of bulk soils (*P*>0.05), but there was significant variation in these indices of the rhizosphere soils. CKR had significantly higher Shannon diversity than LR and HR (*P*<0.05), but not for MR. For the Chao1 richness index, a significant increase was observed with HR relative to that of LR (*P*<0.05) but the increment was not significant for MR (*P*<0.05).

### 3.4 Genera correlated with the P application rate

**Figure 3:**
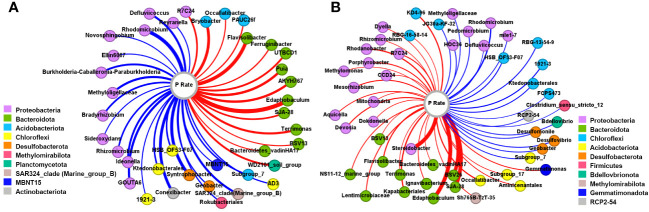
Co-occurrence network between genera and P input rates in the rhizosphere **(A)** and bulk **(B)** soils. The color of the node represents the phylum that the genus belongs to. The red and blue linkages indicate the positive and negative correlations, respectively.

The genera significantly correlated with the P application rate in the rhizosphere (**Figure 3A**) and bulk (**Figure 3B**) soils (*P<*0.05) were examined. In the rhizosphere soils, 38 genera were significantly correlated with P amount, among which 60.5% were negative correlations. Most of the negatively correlated genera belonged to Proteobacteria, then Chloroflexi. Among the 15 positively correlated genera, 10 were affiliated to Bacteroidota and 3 were Acidobacteriota. For bulk soils, 50 genera exhibited a significant correlation with the P application rate, among which there were 31 positive correlations, with 41.94% and 35.48% being Proteobacteria and Bacteroidota, respectively. Negative correlations were mainly observed in the phyla Chloroflexi and Proteobacteria.

### 3.5 The Effects of different P inputs on the relative abundance of genes involved in P transformation

**Figure 4:**
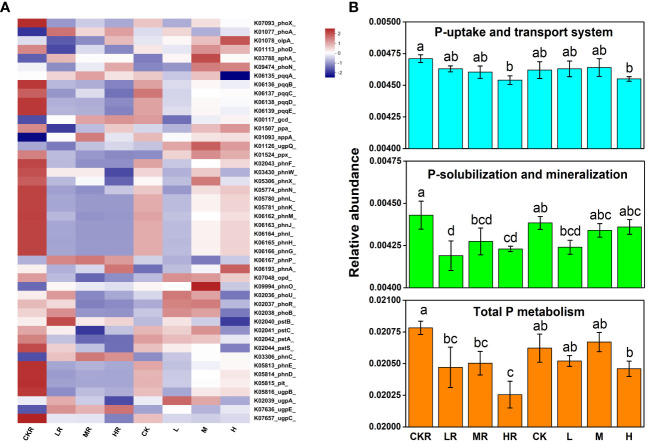
Heatmap of key functional genes involved in P cycling **(A)** and the relative abundance of representative genes responsible for microbial P uptake and transport, Pi solubilization and Po mineralization, and total P metabolism **(B)**. CK, no P fertilization; L, low P input rate; M, medium P input rate; H, high P input rate. R represents rhizosphere soil. Different letters above the error bars indicate significant differences (*P*<0.05).

In comparison with no P treatment, P addition significantly decreased the total P metabolism (*P*<0.05) of the rhizosphere soils, and this was more obvious at high P input (**Figure 4B**). The same trend was observed in the total relative abundance of the genes involved in P solubilization and mineralization (*P*<0.05) (**Figure 4B**), but a significant decrease was only found under high P input for genes involved in P uptake and transport (*P*<0.05) (**Figure 4B**). In contrast, there was no significant difference in the relative abundance of genes among different treatments of bulk soils (*P*>0.05). Different P application rates had no significant effects on the relative abundance of the genes involved in P starvation response regulation in the rhizosphere soils (*P*>0.05), whereas high P input decreased the relative abundances of *phoU* and *phoB* in bulk soils compared with the other two P treatments (*P*<0.05) (**Figure 4A**).

Regarding individual genes involved in inorganic P solubilization, P input significantly decreased the relative abundance of *pqqBCDE* in the rhizosphere soils and bulk soils (*P*>0.05). The same situation was observed in the relative abundance of genes encoding C-P lyases (*phn*). Moreover, bulk soils treated with medium and high concentrations of P had higher relative abundances of *pqq* and *phn* genes compared with the rhizosphere soils (*P*>0.05).

### 3.6 The effects of different P inputs on the relative abundance of genes involved in N-transformation

**Figure 5:**
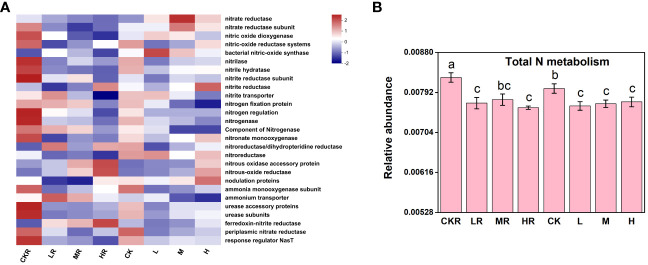
The heatmap of key functional genes involved in the N cycle **(A)** and the relative abundance of representative genes responsible for N metabolism **(B)**. CK, no P fertilization; L, low P input rate; M, medium P input rate; H, high P input rate. R represents rhizosphere soil. Different letters above the error bars indicate significant differences (*P*<0.05).

The addition of P significantly decreased total N metabolism in rhizosphere and bulk soils (*P<*0.05), but no significant variations were observed among the different P treatments (*P*>0.05) (**Figure 5B**). Similar to the effects of P input on individual P transformation function, significant decreases in N transformation function were mostly found in the rhizosphere soils, including among genes encoding nitrite hydratase, a component of nitrogenase, urease accessory proteins, urease subunits, and response regulator NasT (*P<*0.05) (**Figure 5A**). In addition, at the high P input rate, the relative abundance of genes involved in N fixation was decreased in rhizosphere soil, but the difference was not significant (*P*>0.05) P addition significantly decreased total N metabolism in rhizosphere and bulk soils (*P*<0.05), but no significant variations were observed among the different P treatments (*P*>0.05) (**Figure 5B**).

### 3.7 PLS-PM analysis of the effects of different P inputs on soil properties and the bacterial community in rhizosphere and bulk soils

**Figure 6:**
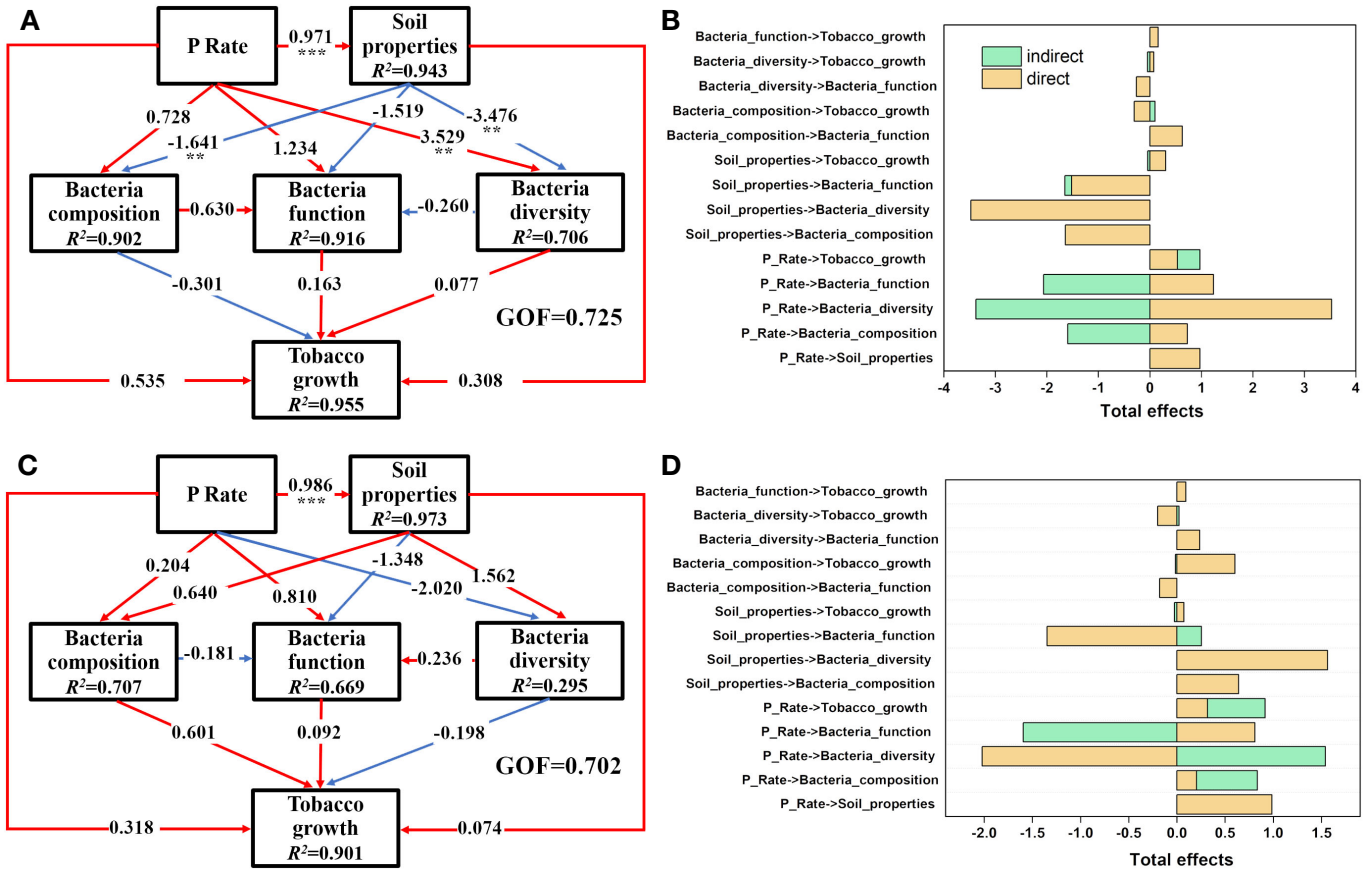
Direct and indirect effects of P input rate, soil properties, bacterial diversity, bacterial composition, and bacterial functions on tobacco growth in rhizosphere **(A, B)** and bulk **(C, D)** soils as illustrated by PLS-PM. Soil properties are latent variables of the measured soil parameters, as shown in **Tables 1**, **2**, and the main soil properties are selected through PLS-PM analysis. Bacterial composition is the first two PCoA axes of bacterial communities and bacterial functions are the abundance of genes involved in P and N metabolism, as shown in **Figures 4B**, **5B**, respectively. Numbers on the arrowed lines indicate the normalized direct path coefficient. R^2^ below the latent variables represents the variations of dependent variables explained by the inner model. The GOF index represents the goodness of fit. Asterisks represent significant effects: **, *P*<0.01; ***, *P*<0.001.

PLS-PM analysis was performed separately to elucidate the specific causality among the P input rate, soil properties, bacterial community, and tobacco growth in the rhizosphere (**Figures 6A, B**) and bulk (**Figures 6C, D**) soils. In the rhizosphere soils, P input directly and significantly induced changes in the soil properties (*P*<0.05) and then negatively impacted soil bacterial composition (mainly PAC, AP, and Fe-P) and diversity (mainly AN and ACP) (*P*<0.05). P input also directly impacted the soil rhizospheric bacterial community, with a significant effect observed on bacterial diversity (path coefficient =3.529) (*P*<0.05). Those pathways explained 90.2%, 91.6%, and 70.6% of the total variance in soil bacterial composition, function, and diversity (**Figure 6A**). Moreover, the results indicate that P input directly and positively affected the soil bacterial community, which was opposite to the behavior of soil properties. In the P addition experiment, P input plus the corresponding variations of rhizosphere soil properties and bacterial community contributed 95.5% of the total variance in tobacco growth. Congruent with the situation in the rhizosphere soils, P application also significantly changed the bulk soil properties directly (*P*<0.05); however, P application had negative effects on bacterial diversity and positive effects on composition and function, respectively (*P*>0.05), in the bulk soils, which was in contrast to the situation in the rhizosphere soils (**Figure 6C**).

## 4 Discussion

### 4.1 The effects of P application at different rates on N/P cycling and plant growth

P, which is a non-renewable nutrient, plays an essential role in plants, but the majority of applied P fertilizer is rapidly immobilized by soil fixation and becomes inaccessible for plant uptake. For instance, in highly weathered tropical soils, such as ferralsol, P limitation is often the main constrain for plant growth (Ehlers et al., 2010) because P is readily immobilized by fixation as Al/Fe oxides/hydroxides or precipitation as poorly utilized Fe-P and Al-P (Liu et al., 2019). A ^33^P-labeling experiment conducted on two acidic soils revealed that a large amount of added P was stored in the abiotic P pools and was not recovered in the labile P fraction or the biotic pools (Spohn et al., 2018). In the sampled soils in this study, Fe-P was the most abundant P fraction and increased with added P fertilizer (**Table 2**), indicating unsaturated soil P adsorption capacity. By contrast, a significantly decreased biomass of tobacco was observed under conditions of no P and low P (**Table 1**), suggesting P limitation of plant growth. Consistently, fitting results revealed a more obvious increase of tobacco biomass production with the increased P application rate (**Supplementary Figure 1**), coinciding with previous studies (Jiang et al., 2019; Hou et al., 2020). However, considering the sharp P adsorption of the target soils, applying a large amount of one-time base P fertilizer is inadvisable and more attention should be directed at identifying the optimal amount and time to employ P fertilizer.

N is another essential nutrient for plant growth, participating in photosynthesis (Liu et al., 2019). Generally, N and P strongly interact with each other in biogeochemical processes, and this interaction is usually synergistic based on the nutrient stoichiometric homeostasis theory (Cleveland and Liptzin, 2007). According to the literature, P application can accelerate N cycling by stimulating plant N uptake and soil N transformation. However, the response of plant aboveground biomass-N (AGB-N) to P addition depends on the vegetation characteristics, with woody plants showing decreased AGB-N and herbaceous plants showing increased AGB-N (Xia et al., 2023). Our results conform with these findings and reveal that P application increased N and P uptake in tobacco (**Table 1**), an annual herb. The significant decrease in soil TN with MR treatment may be ascribed to the more coordinated ratio of nitrogen to phosphorus in this treatment (Dai et al., 2020), which is more conducive to microbial activity. This could also explain the relatively higher Shannon diversity observed in MR compared with LR and HR samples. Additionally, the relatively lower leaf N concentration at high P input may be ascribed to a dilution effect as the AGB was increased (Jiang et al., 2019). The increased N uptake of tobacco plants further led to the decreased TN concentrations of the rhizosphere soils with P applied (**Table 1**).

Apart from P mobilization, microorganisms also assimilate P to build cellular and genetic components and perform metabolic and energy transfer processes (Sosa, 2018). In addition, microbial biomass P contributes a substantial fraction of the total soil P, which can be an important active P reservoir (Zederer et al., 2017; Spohn et al., 2018) considering its fast turnover rate (Achat et al., 2012). In soils, the interactions of nutrient N and P are complicated and are mediated by the soil stoichiometric ratio of N and P (N:P) (Cleveland and Liptzin, 2007; Brown et al., 2022). Generally, low levels of N addition stimulate phosphatase activity and increase P availability, favoring plant C assimilation, whereas at high levels of N addition, the demand for P is also increased and may not be satisfied by the N-induced P availability increase, subsequently resulting in P limitation for plant growth (Liu et al., 2019; Peng et al., 2019).

### 4.2 The effects of P application on the soil microbial community

Soil microorganisms and plant roots are key regulators of the biogeochemical cycling of P and have developed several mechanisms to solubilize bound Pi and mineralize Po by releasing organic acid anions/protons (Smits et al., 2012; Barrow and Lambers, 2022) and extracellular phosphatases (Nassal et al., 2018), respectively. Therefore, the rhizosphere is a hotspot for soil microbial activity, as the root-derived organic C stimulates the growth and metabolic processes of soil microbes (Chen et al., 2002; Jones et al., 2009). Similar to the previous findings, our results displayed marked variations in the rhizosphere soils with respect to the bacterial community structure, diversity, P metabolism, and so on. As shown in **Figure 6**, the impact of different P input rates on rhizosphere soil bacterial diversity and P metabolic function was more profound than on bulk soils. This may be due to microorganisms being more active in the rhizosphere region and then competing with the roots for P, especially under the condition of no P application (Marschner et al., 2011). We also found that Nitrospirota was increased in the rhizosphere soils, especially under low and medium P input rates, indicating that the addition of P increases N metabolism. Nitrospirota is reported to participate in N-based metabolisms, likely nitrite oxidation and complete ammonia oxidation (comammox), as well as manganese oxidation (D’Angelo et al., 2023). Zhu et al. (2022) found that Nitrospirota is a key taxon in regulating rice root N uptake under abrupt drought flood alternation. Desulfobacterota was also increased in the rhizosphere soils, and bacteria of this phylum are known to have various metabolic abilities, including sulfate respiration, aliphatic and aromatic hydrocarbon degradation, nitrogen fixation, denitrification, organohalide respiration, and dissimilatory iron reduction (Langwig et al., 2022).

Soil P availability has been reported to influence the structure of soil microbial communities, as well as the corresponding functions and metabolisms (Liu et al., 2022; Wu et al., 2023). Consequently, different fertilization regimes exert different effects on microbial life strategies, shaping soil P cycling processes (Fierer et al., 2007; Bergkemper et al., 2016). In this study, the addition of P decreased the relative abundance of Proteobacteria in rhizosphere soils but increased the proportion of Bacteroidota (*P*<0.05) (**Figure 1A**). The same results were also observed by Zhang et al. (2023a) in a P addition pot experiment with loess soil. These results are contrary to the general perception, as Proteobacteria are copiotrophic microorganisms that always exhibit high growth rates when resources are abundant. Thus, copiotrophs are usually stimulated following the input of nutrients, such as N and P (Fierer et al., 2007; Dai et al., 2020). Regarding our inconsistent results, one possible explanation is that although an enormous number of bacteria are classified in the same phylum, these bacteria have their own diverse phylogeny and physiology, and some may not share the common ecological characteristics, e.g., life history (Fierer et al., 2007). In this study, the higher abundance of Proteobacteria in the no P treatment may be ascribed to the increase of P-solubilizing and -mineralizing microorganisms, most of which belong to the phylum Proteobacteria (Wei et al., 2019). With respect to Bacteroidota, the increased relative abundance may relate to the increased exudates of rhizosphere soils, especially at the high P level, as these bacteria are reported to be important carbohydrate degraders by secreting diverse sets of carbohydrate-active enzymes (Kruczynska et al., 2023; Zhang et al., 2023b). Moreover, other bacterial phyla may also respond to P input, as in other research, the addition of P significantly increased only two phyla, Armatimonadetes and Chlorobi (Ling et al., 2017). In addition, at the order level, P fertilization significantly decreased the abundance of Burkholderiales and Rhizobiales, which are reported to participate in P immobilization (Zheng et al., 2018).

### 4.3 The effects of P application on soil microbial N/P metabolism

Soil microorganisms act as a sink and source of available P by mediating key processes in nutrient biogeochemical cycling (Ehlers et al., 2010), including P starvation response regulation (*phoU*, *phoR*, and *phoB*), P uptake and transport (*pst* and *pit*), Pi solubilization (*pqq* and *gcd*), and Po mineralization (*phoD*, *phoA*, *phoX*, *phoN*, *appA*, and *phn*) (Dai et al., 2020). Our results revealed that P application significantly decreased soil total N metabolism (rhizosphere and bulk soils) (**Figure 5B**) and total P metabolism (only rhizosphere soils) (**Figure 4B**) (*P*<0.05). Consistently, significantly increased nitrous oxide (N_2_O) emissions were found in P-limited forest soils following N addition, inferring stimulated N immobilization and the metabolism of microbes (Mehnaz et al., 2019). The decreased N metabolism in the soils with P added may be attributed to the decreased N substrates due to their uptake by tobacco. This is supported by our observation that the TN concentrations of the soils with P added were lower than those with no P treatment (**Table 1**). Similarly, Baral et al. (2014) observed decreased N_2_O emissions due to increased N uptake by maize. Nevertheless, with the medium and high level of P, the N_2_O reduction enzymes were increased in this study. This could be explained by the addition of P increasing the activities of heterotrophic microorganisms and hence increasing organic matter decomposition and oxygen consumption (Mori et al., 2013), favoring the reduction of N_2_O to dinitrogen (N_2_) (Zhou et al., 2018). The effects of P addition on microbial P metabolism can be explained by the incorporation of nutrient P resulting in a rich soil P concentration, which weakens the important roles of plants and microbes in P supply. This is supported by the increased microbial P immobilization under conditions of low P (Spohn et al., 2018).

Regarding phosphatase activity, inconsistent findings have been reported in the literature on the responses to P fertilization. For instance, significantly decreased ALP and urease activities were observed when adding P (Zhang et al., 2023c), whereas other studies have reported increased ALP and ACP activities (Ikoyi et al., 2018) or no variation in ALP activity with increasing the addition of P (Fraser et al., 2015). In this study, P fertilization also had no significant impact on soil ALP and ACP activities (**Table 1**), but significantly increased phytase activity was detected in response to the increasing P application rate. The reasons for our findings may be that microbes are inclined to utilize recently formed organic P first, rather than more distantly formed bound P (Ikoyi et al., 2018), as is the case for sulfur cycling (Ghani et al., 1993). Specifically, P application may have induced higher immobilization rates immediately, then higher remineralization rates of the recently formed organic P occurred, resulting in higher phytase activity.

Phosphonates are a class of organophosphorus compounds characterized by a stable C-P bond, presenting as valuable P sources for microorganisms. C-P lyases (encoded by *phn* genes) and phosphonatase can cleave the C-P bond and help soil microbes utilize phosphonates effectively (Huang et al., 2005). Moreover, C-P lyases and phosphonatase are reportedly expressed under the condition of P starvation (Obojska and Lejczak, 2003). Congruently, Hu et al. (2023) found decreased *phnC* gene abundance after long-term manure input. In our study, changes in the relative abundance of *phn* genes were different between rhizosphere and bulk soils (**Figure 4A**). In the rhizosphere soils, only the no P treatment showed a rich abundance of *phn* genes, indicating the condition of P starvation for microbes. Furthermore, we could speculate that all P amendment treatments, regardless of P input amount, were not P limiting conditions for microbes in tobacco rhizosphere. On the contrary, in bulk soils, all treatments except the low P application rate had an increased relative abundance of *phn* genes, indicative of the P-limiting condition of bulk soils for microbes. This also underlines the importance of interactions between plant roots and the accompanying microbes in increasing P supply. Finally, the decreased *phn* genes in the bulk soils amended with low P might also be ascribed to the relatively higher ALP activities (**Table 1**), which play the same role in Po mineralization and could therefore compensate for the function.

## 5 Conclusion

In this study, the addition of P—regardless of input rate—increased tobacco growth, and the amplitude also increased with ascending P application, indicating that P limitation occurred during the early growth of the tobacco seedlings. This observation is ascribed to the vigorous P adsorption effects of the investigated soils, which were rich in Al and Fe. Indeed, Fe-P and Al-P were the dominant P fractions in the sampled soils and increased with the increased P amendment. Thus, although P was abundant in the soils, it was only sparingly available to plants. PLS-PM revealed that the addition of P significantly influenced soil properties (*P*<0.001), consequently affecting soil bacterial composition and diversity, especially for the rhizosphere soils (*P*<0.01). Moreover, P application significantly decreased microbial P and N metabolism in rhizosphere soils (*P*<0.05). Accordingly, the high P condition was limiting for plants, not for microbes, which is not conducive to the role of microorganisms in promoting nutrient cycling. Thus, more attention should be paid to motivating potentials of soil functional microbes in P cycling under conditions of surplus P, especially at the beginning of plant growth when the roots are underdeveloped and unable to absorb enough nutrients. In summary, this study highlights the importance of stimulating P cycling microorganisms to promote P uptake by plants, rather than through the continual application of P. Our findings provide ideas for improving the utilization efficiency of P in the early stage of tobacco growth in P-rich soils.

## Data availability statement

The datasets presented in this study can be found in online repositories. The names of the repository/repositories and accession number(s) can be found below: BioProject, PRJNA1031332.

## Author contributions

BZ: Writing – original draft, Visualization, Investigation. SC: Writing – review & editing, Methodology. SP: Writing – review & editing, Visualization, Software. WL: Writing – review & editing, Funding acquisition. CL: Writing – review & editing, Funding acquisition. QW: Writing – review & editing, Investigation. YW: Writing – review & editing, Supervision, Conceptualization. JG: Writing – review & editing, Conceptualization.

## Supplementary material

SUPPLEMENTARY FIGURE 1 Correlation between P input rates and the tobacco biomass.
